# A Call to Analyze Sex, Gender, and Sexual Orientation in Psychopathology Research: An Illustration with ADHD and Internalizing Symptoms in Emerging Adults

**DOI:** 10.1007/s10862-024-10188-3

**Published:** 2025-02-03

**Authors:** Cynthia M. Hartung, Elizabeth K. Lefler, Tamara M. Abu-Ramadan, Anne E. Stevens, Judah W. Serrano, Emily A. Miller, Christopher R. Shelton

**Affiliations:** 1 University of Wyoming, Laramie, WY USA; 2 University of Oregon, Portland, OR USA; 3 Children’s National Hospital, Washington, DC, USA; 4 University of Denver, Denver, CO USA; 5 Pennsylvania State University, The Behrend College, Erie, PA USA

**Keywords:** Sexual and gender minorities, ADHD, Depression, Anxiety, Emerging adults

## Abstract

We have historically ignored sex/gender and conducted sex- and gender-neutral psychopathology research. There is a clear need to analyze potential differences and similarities between individuals with various sexes, genders, and sexual orientations in psychopathology research. Specifically, we need to stop ignoring sex, gender, and sexual orientation, conduct analyses that go beyond the binary, and analyze these important variables for generalizability even when the primary research question is not about sex, gender, and sexual orientation. In the current study we examined ADHD and internalizing symptoms in a community sample to compare different ways to analyze data and better understand differences and similarities across groups. We predicted that a richer understanding of sex/gender differences would emerge when we compared sex and gender minority (SGM) participants to cisgender heterosexual women (CHW) and men (CHM) rather than conducting binary analyses. Emerging adults (*N* = 2,938; ages 18–29 years) completed an online survey, responding to demographic items, as well as ADHD and internalizing symptoms. Binary analyses using biological sex and gender identity yielded no differences in ADHD symptoms, and the expected female preponderance in internalizing symptoms. However, when analyzed across three groups, individuals in the SGM group reported higher levels of ADHD and internalizing symptoms compared with the other two groups. Notably, no differences emerged for internalizing symptoms across CHW and CHM when the SGM group was included. This is compelling evidence that analyzing sex, gender, and sexual orientation more systematically and precisely in psychopathology research is warranted.

Historically, psychopathology researchers have given short shrift to understanding differences (and similarities) between men’s and women’s experiences of mental illness (Hartung & Widiger, 1998; Hartung & Lelfer 2019; Howard et al., 2017). Hartung and Lefler (2019) argued that studies of mental illness have suffered from a type of sampling bias: the tendency to select samples composed of only those most prototypical of a disorder (e.g., studies of only boys with autism), leading to diagnostic criteria/symptoms, theories of etiology, and treatment strategies that may only pertain to one group or may be more relevant for one group. There has also been a tendency to conflate sex and gender terms in research studies (Hartung & Lefler, 2019; Howard et al., 2017), leading to: (a) unclear conclusions about how biological/genetic and/or societal/environmental roles contribute to the development of mental illness and differential sex/gender prevalence rates; and (b) unclear treatment recommendations for diverse individuals, among other concerns. When researchers do include both men and women or boys and girls (which they are doing at increasing rates), data analytic bias remains (i.e., a bias toward not running analyses to compare sex or gender groups; Hartung & Lefler, 2019). Furthermore, when sex/gender are considered in psychopathology research, which is not often, a binary approach is almost always used. When the binary approach is used, sex/gender are often conflated, as mentioned earlier, and this approach systematically ignores the experiences of sexual and gender minorities (SGM^1^; i.e., lesbian, gay, bisexual, transgender, queer [LGBTQ +]; National Institutes of Health [NIH], 2024).

For example, Hartung and Lefler (2019) reviewed all studies published in the *Journal of Abnormal Psychology* (now the *Journal of Psychopathology and Clinical Science*) from 2010 to 2017 and found that while 80% of studies included both males and females, only 41.7% of those study authors conducted analyses to determine whether their findings generalized to both binary sex groups (i.e., analyzed sex/gender as an independent, moderator, or mediator variable) whereas 44.4% of authors ignored sex/gender in the preliminary and primary analysis, and 13.8% of authors included sex/gender as a covariate which ignores sex/gender rather than helping us determine whether our findings apply to both men and women. This is not even to mention the erasure of non-binary, transgender, intersex, and queer people from psychopathology research entirely. Very few studies, with the exception of those directly studying LGBTQ + experiences, analyze results with these groups in mind, let alone list rates of these identities in their participants' sections. The problem is far-reaching; when we fail to understand psychopathology in girls/women, boys/men, and individuals with diverse sexual and gender identities, we will fail to accurately and appropriately diagnose and treat them.

It is clear that we need to stop ignoring sex/gender in psychopathology research. However, possible approaches to analyzing sex, gender, and sexual orientation (S/G/SO) have not been extensively explored. Thus, in the current paper we use attention-deficit/hyperactivity disorder (ADHD) and internalizing symptoms to call attention to this problem and explore analytic options for researchers to better capture potential S/G/SO differences (and similarities) in their studies. Prior to describing our current study in more detail, we briefly discuss what we know about S/G/SO differences and similarities in ADHD and internalizing symptoms.

## Sex, Gender, and Sexual Orientation in ADHD and Internalizing Symptoms

Both ADHD and internalizing symptoms (i.e., depression and anxiety symptoms) have long-established sex/gender differences. Per the *Diagnostic and Statistical Manual of Mental Disorders, Fifth Edition, Text-Revision* (*DSM-5-TR*; American Psychiatric Association [APA], 2022), ADHD shows a male preponderance for adults of 1.6:1, major depressive disorder (MDD) shows a female preponderance of 1.5:1 to 3:1, generalized anxiety disorder (GAD) a female preponderance of 2:1, and panic disorder a female preponderance of 2:1. These numbers are supported by various worldwide meta-analyses and large-scale studies of sex/gender differences in these common disorders. For example, Salk et al. (2017) conducted a worldwide meta-analysis of 95 studies which confirmed that women self-reported higher levels of depression symptoms than men. This sex/gender difference was shown to peak in adolescence, decline in emerging adulthood, and remain relatively stable throughout adulthood. Next, Willcutt (2012) conducted a worldwide meta-analysis that included 96 studies. This analysis concluded that the ratio of ADHD in adults was 1.6 M:1F. More recently, a review by Hinshaw et al. (2022) suggested that the rates of ADHD in adults might be less discrepant and approaching a 1:1 ratio. Finally, in a study of over 20,000 participants (i.e., Collaborative Psychiatric Epidemiology Study [CPES]) McLean et al. (2011) concluded that the lifetime female:male prevalence ratio of any anxiety disorder to be 1.7F:1 M, and found that women reported higher illness burdens than men. This suggests that women outnumber men in anxiety diagnoses, and may also be more impaired by their anxiety. In all, these studies tell us that ADHD and several common internalizing disorders have well-documented sex/gender differences. While this amount of empirical data on sex/gender differences in prevalence rates is heartening, it is nonetheless limited. Indeed, these studies may have conflated and/or poorly operationalized sex and gender on their demographics forms, few studies account for LGBTQ + experiences, and it is still not the norm for research teams to include any analyses that consider sex, gender, or sexual orientation.

Data on SGM mental health has focused on internalizing symptoms, substance use, and suicide risk (e.g., Marshal et al., 2011). For example, in a meta-analysis of depression and anxiety, Ross et al. (2018) found that heterosexual/straight-identified individuals had the lowest rates of depression/anxiety, LGB-identified people had higher rates, and bisexual participants reported similar or even higher rates than lesbian and gay participants. Moreover, Borgogna et al. (2019) found that trans and gender non-conforming (TGNC) college students had higher rates of depression and anxiety than cisgender participants. The authors also noted that those in emerging identity categories (e.g., demisexual, asexual, TGNC) reported the highest rates of depression and anxiety. Less research has been conducted on the rates of ADHD in SGM populations. In one study by Dawson et al. (2017), transgender participants had higher rates of ADHD (in addition to anxiety and depression) as compared to cisgender participants. Likewise, Strange et al. (2014) found that children with an ADHD diagnosis were 6.64 times more likely than controls to have their parents indicate some gender variance on their behalf. While this area of research is growing, there is still much to learn.

It is important to study SGM samples because of these higher rates of disorders. Indeed, minority stress theory states that individuals with a minoritized identity (or more than one such identity) are subject to micro- and macro-aggressions that result in stress, and confer additional risk for mental health concerns (Meyer, 2003). This may also lead to internalized homophobia and/or transphobia (Hendricks & Testa, 2012; Herek et al., 1997) which can cause people in the LGBTQ + community to harbor negative beliefs about their own sexual or gender minority status, which can in turn lead to additional risk for internalizing problems (Newcomb & Mustanski, 2010). This population is also important to understand because more and more people are identifying in these ways (Lefler et al., 2023). In fact, 22.7% of emerging adults (in this case defined as ages 18 to 25 years) reported an SGM identity as compared to 1.3% of older adults (ages 65 to 84 years; Lefler et al., 2023). Taken together, there is a need for additional research on emerging adult mental health, with an eye toward better understanding S/G/SO.

## The Current Study

There is a clear need for psychopathology research that directly investigates potential differences and similarities between individuals with various sexes, genders, and sexual orientations. Progress has been made in recent decades in that more and more studies include individuals from “both” binary sex/gender categories. However, this is still limiting for several reasons as outlined above. What is needed next is more S/G/SO research, less focus on only the binary, and more studies that examine these important variables even when these identities are not the central research question. To this end, in the current study we examined ADHD and internalizing symptoms in a community sample to illustrate the importance of finding creative ways to analyze data and better understand differences and similarities across groups. We chose to focus on emerging adults given that this group is more likely to endorse SGM identities than other groups of adults (Lefler et al., 2023). Specifically, our research question was whether established sex/gender differences would change if SGM participants were removed from these binary groups and analyzed separately. We predicted that a richer understanding of sex/gender differences would emerge when we carefully examined SGM participants and compared them to cisgender heterosexual women (CHW) and men (CHM). In the current study, we used a large emerging adult sample (2,900 +) to examine this research question. We conducted analyses on broad internalizing symptoms, which might be more obviously elevated for SGM participants, but also ADHD symptoms, which have been under-studied in this population.

## Method

### Participants

Participants were 2,938 emerging adults who resided in the United States and completed an online survey via Amazon’s Mechanical Turk (MTurk; https://www.mturk.com). Participants ranged in age from 18 to 29 years (*M* = 24.77, *SD* = 3.03) and provided demographic information regarding age, biological sex, gender identity, sexual orientation, race/ethnicity, and highest level of education completed.

First, we describe participants in terms of their self-reported biological sex, gender identity, and sexual orientation. For biological sex, participants endorsed the following: female (*n* = 1,801; 61.3%), male (*n* = 1,078; 36.7%), intersex (*n* = 54; 1.8%), not listed/missing (*n* = 5; 0.1%). Next, for gender identity, participants endorsed the following: female/woman (*n* = 1,706; 58.1%), male/man (*n* = 1,059; 36.0%), non-binary/fluid queer/gender queer (*n* = 143; 4.9%), not sure/exploring (n = 16; 0.5%), not listed/missing (*n* = 14; 0.5%). Finally, for sexual orientation, participants endorsed the following: heterosexual/straight (*n* = 2,084; 70.9%), gay/lesbian (*n* = 194; 6.6%), bisexual (*n* = 410; 14.0%), queer (n = 48; 1.6%), pansexual (n = 90, 3.1%), asexual (n = 41, 1.4%), not sure/exploring (n = 56; 1.9%), not listed/missing (*n* = 15; 0.5%).

Next, we describe participants in terms of race/ethnicity, level of education, and self-reported lifetime diagnosis of ADHD, depression, or anxiety. For race/ethnicity, participants responded in the following ways: Asian/Asian American (*n* = 377; 12.8%), Black or African American (*n* = 322; 11.0%), Latinx/Hispanic (*n* = 316; 10.8%), Native American/American Indian/Alaska Native/Indigenous (*n* = 59; 2.0%), Middle Eastern/North African (*n* = 28; 1.0%), Pacific Islander/Native Hawaiian (*n* = 12; 0.4%), White (*n* = 1,744; 54.4%), multiracial (n = 70; 2.4%), and not listed /missing (*n* = 10; 0.3%). For level of education, participants endorsed the following: doctoral degree (*n* = 46; 1.6%), master’s degree (*n* = 327; 11.1%), bachelor's degree (*n* = 1,123; 38.2%), associate’s degree (*n* = 398; 13.5%), high school diploma or GED (*n* = 1021; 34.8%), less than high school or GED (*n* = 16; 0.5%), and prefer not to answer, not listed, or missing (n = 7; 0.3%). In terms of self-reported lifetime diagnosis (for our variables of interest), our participants reported ADHD (*n* = 552; 18.8%), depression (*n* = 1,016; 34.6%), and anxiety (*n* = 1,286; 43.8%. Additionally, the sample as a whole had an average of 18.03 (*SD* = 11.81) for ADHD severity score, and an average of 24.33 (*SD* = 15.18) for internalizing total score (more on how these were calculated in Measures below).

### Measures

The *Depression, Anxiety, and Stress Scale* (DASS; Lovibond & Lovibond, 1995) is a 21-item self-report measure designed to assess symptoms of depression, anxiety, and stress. Individuals reported the extent to which they experienced each symptom in the past week using a four-point Likert scale (0 = *Never* to 3 = *Almost Always*). The DASS produces a total score and three subscale scores. This measure has been shown to have acceptable to excellent reliability and validity (Antony et al., 1998) as evidenced by strong convergent validity and the ability to distinguish features of depression and anxiety. In the current study, we used the total score ranging from 0 to 63 as a measure of overall internalizing symptoms, internal consistency was excellent (α = 0.95).

The *Diagnostic and Statistical Manual of Mental Disorders-Fifth Edition* (*DSM-5*) ADHD symptom checklist is a self-report measure containing the 18 ADHD items from the *DSM-5* (American Psychiatric Association [APA], 2013). This measure includes 9 inattention and 9 hyperactivity/impulsivity items. Participants indicated whether they experienced each item on a 4-point Likert scale (0 = *Never or rarely* to 3 = *Very often*) based on the past six months. By virtue of this list of items being pulled directly from the *DSM-5*, it is considered a valid measurement of *DSM-5*-defined ADHD. For the current study, a total severity score was calculated ranging from 0 to 54, and internal consistency was excellent (α = 0.93).

### Procedure

All study procedures were approved by the Institutional Review Board at the first author’s university. Participation in this study occurred through MTurk, an online crowdsourcing platform. Participants completed an online survey created using the Qualtrics Research Suite. After providing informed consent, participants completed the demographic items, the *DSM-5* ADHD Checklist, and the DASS-21. The survey consisted of a total of 56 items, including two attention check items, and was took 3–5 min to complete. When a participant failed either attention check item (2.5%), their participation was automatically discontinued. Participants who completed the study were compensated with $0.20, which is considered fair compensation for MTurk surveys of this length (Moss et al., 2023).

### Data Preparation & Analytic Plan

Prior to analysis, the dataset was screened for missing data, duplicate participation, and for potential issues with univariate normality and outliers. Data from participants who had any data missing on the variables of interest was deleted from the dataset. The final sample was *N* = 2,938. For the current study, we compared levels of ADHD and internalizing symptoms across two sex/gender groups and three sex/gender/sexual orientation groups. We conducted two, two-group analyses; one for biological sex and one for gender identity. For our first two-group analysis, we compared biological females (*n* = 1,801) to biological males (*n* = 1,078; Table 1), and for our second two-group analysis we compared those who identified as woman/female (*n* = 1,706) to those who identified as men/male (*n* = 1,059; Table 2). For the three-group approach, we compared cisgender heterosexual women (CHW; *n* = 1,168), cisgender heterosexual men (CHM; *n* = 895), and a sexual and gender minority (SGM; *n* = 869) group. The SGM group consisted of individuals who endorsed: (a) intersex on the biological sex item; (b) non-binary/fluid queer/gender/queer, not sure/exploring, or not listed/missing on the gender identity item; (c) gay/lesbian, bisexual, queer, pansexual, asexual, not sure/exploring, or not listed/missing on the sexual orientation item; (d) female on the biological sex item and male on the gender identity item; or (e) male on the biological sex item and female on the gender identity item. When three group analyses were significant, we conducted pairwise post-hoc Games-Howell analyses. This procedure was chosen because the equal variance assumption was violated in some of these analyses. Power analyses conducted using G-Power 3.1.9.7 (Faul et al., 2009) indicated that at least 64 participants per group were needed to achieve adequate power (0.80) to detect a medium effect (*d* = 0.50) with a standard significance level (*p* = 0.05) using independent samples* t*-tests. For one-way ANOVAs with three groups, at least 80 participants per group were needed to achieve adequate power (0.80) to detect a medium effect (*f* = 0.25) with a standard significance level (*p* = 0.05). Thus, all of our analyses were adequately powered to detect medium effects.

**Table 1:** Two-Group Analysis: Biological Sex

	Females		Males		*t*	*p*	Cohen’s *d*
	*n* = 1,801		*n* = 1,078		(1, 2877)		
	*M*	*SD*	*M*	*SD*			
ADHD dimension	18.04	11.94	17.66	11.49	0.84	.401	-.03 (-.04-.11)
Internalizing dimension	25.16	15.20	22.47	15.00	4.62	< .001	-.18 (.10-.25)

*Notes. N* = 2,879; ADHD range = 0–54; Internalizing range = 0–63; For Cohen’s d, 0.2 to 0.4 is a small, 0.5 to 0.7 is a medium, and >= 0.8 is a large effect. The sample size for this table is smaller than the full sample because only binary respondents were included

**Table 2:** Two-Group Analysis: Gender Identity

	Women		Men		*t*	*p*	Cohen’s *d*
	*n* = 1,706		*n* = 1,059		(1, 2763)		
	*M*	*SD*	*M*	*SD*			
ADHD dimension	17.73	11.89	17.66	11.57	0.16	.872	-.01 (-.07-.08)
Internalizing dimension	24.67	15.12	22.44	15.11	3.77	< .001	-.15 (.07-.22)

*Notes. N* = 2,879; ADHD range = 0–54; Internalizing range = 0–63; For Cohen’s d, 0.2 to 0.4 is a small, 0.5 to 0.7 is a medium, and >= 0.8 is a large effect. The sample size for this table is smaller than the full sample because only binary respondents were included

## Results

### Two-Group Analyses

#### Biological Sex

We first conducted two independent samples *t*-tests to examine potential differences by biological sex in ADHD and internalizing symptoms based on self-report (Table 1). For ADHD symptoms, the mean for biological females was 18.04 (*SD* = 11.94), and the mean for biological males was 17.66 (*SD* = 11.49). This difference was not statistically significant (*t* = 0.84, *p* = .401, *d* = 0.03). For internalizing symptoms, the mean for biological females was 25.16 (*SD* = 1.36), and the mean for biological males was 22.47 (*SD* = 15.00). This difference was statistically significant (*t* = 4.62, *p* < .001, *d* = 0.18) and approaching a small effect with females reporting more internalizing symptoms than males.

#### Gender Identity

Next, we conducted two independent samples *t*-tests to examine potential differences by gender identity in ADHD and internalizing symptoms based on self-report (Table 2). For ADHD symptoms, the mean for those who identified as women was 17.73 (*SD* = 11.89), and the mean for those who identified as men was 17.66 (*SD* = 11.57). This difference was not statistically significant (*t* = 0.16, *p* = .872, *d* = 0.01). For internalizing symptoms, the mean for those who identified as women was 24.57 (*SD* = 15.12) and those who identified as men was 22.44 (*SD* = 15.11). This difference was statistically significant (*t* = 3.77, *p* < .001, *d* = 0.15) and approached a small effect, with women reporting more internalizing symptoms than men.

### Three-Group Analyses

We conducted two one-way univariate analyses of variance tests (i.e., 3 × 1 ANOVAs) to examine potential sex/gender/sexual orientation differences in ADHD and internalizing symptoms (Table 3). For ADHD symptoms, the mean for CHW was 15.93 (*SD* = 11.61), the mean for CHM was 16.72 (*SD* = 11.24), and the mean for SGM was 22.19 (*SD* = 11.59). This difference was statistically significant (*F* = 82.33, *p* < .001, partial η^2^ = 0.05) and corresponded to a small effect. Post-hoc Games-Howell paired comparisons demonstrated that there was no significant difference between CHW and CHM (*p* = .260); however, there were significant differences between CHW and SGM (*p* < .001) and between CHM and SGM (*p* < .001) such that the SGM group reported higher ADHD symptoms.

**Table 3:** Three-Group Analysis: Sex/Gender/Sexual Orientation

	CHW n = 1,168		CHM n = 895		SGM n = 869		*F* (2, 2929)	*p*	Partial η^2^
	*M*	*SD*	*M*	*SD*	*M*	*SD*			
ADHD dimension	15.93^a^	11.61	16.72^a^	11.24	22.19^b^	11.59	82.33	< .001	.05 (.04-.07)
Internalizing dimension	22.20^a^	14.81	21.15^a^	14.87	30.50^b^	14.18	110.60	< .001	.07 (.06-.09)

*Notes. N* = 2,932; CHM = cisgender heterosexual men; CHW = cisgender heterosexual women; SGM = sexual and gender minority; ADHD range = 054; Internalizing range = 0–63; For partial eta squared, .01 to .05 is a small, .06 to .13 is a medium, and > = .14 is a large effect. Intersex individuals are included in the SGM group

For internalizing symptoms, the mean for CHW was 22.20 (*SD* = 14.81), the mean for CHM was 21.15 (SD = 14.87), and the mean for SGM was 30.50 (*SD* = 14.18). This difference was statistically significant (*F* = 110.60, *p* < .001, partial η^2^ = 0.07) and corresponded to a medium effect. Post-hoc Games-Howell paired comparisons demonstrated that there was no significant difference between CHW and CHM (*p* = .247); however, there were significant differences between CHW and SGM (*p* < .001) and between CHM and SGM (*p* < .001) such that the SGM group reported higher internalizing symptoms.

### Exploratory Analyses: Four-Group Analyses

Given that the SGM group reported much higher levels of ADHD and internalizing symptoms than either of the cisgender heterosexual groups, we conducted additional exploratory analyses to examine subgroups of individuals within the SGM group. For these four-group analyses, we compared participants who identified as: (a) cisgender and heterosexual (men and women combined due to no significant differences in previous analyses; *n* = 2,317), (b) cisgender and gay/lesbian (*n* = 141), (c) cisgender and bisexual (*n* = 365), and (d) TGNC and any sexual orientation (*n* = 115). There were two reasons that we did not divide the TGNC group into smaller groups based on sexual orientation. First, the distinction between heterosexual and gay/lesbian is based on a binary that does not apply to those who identify as non-binary. Second, we conducted an additional power analysis for one-way ANOVAs with four groups. We found that at least 90 participants per group were needed to achieve adequate power (0.80) to detect a medium effect (*f* = 0.25) with a standard significance level (*p* = .05) Table 4.

**Table 4:** Four-Group Analysis: Sex/Gender/Sexual Orientation

	Cisgender + Heterosexual *n* = 2,317		Cisgender + Gay/Lesbian *n* = 141		Cisgender + Bisexual * n* = 365		TGNC + Any SO *n* = 115		*F* (3, 2934)	*p*	Partial η^2^
	*M*	*SD*	*M*	*SD*	*M*	*SD*	*M*	*SD*			
ADHD dimension	16.88^a^	11.60	21.92^b^	11.86	22.10^b^	11.67	23.48^b^	11.12	36.28	< .001	.04 (.02-.05)
Internalizing dimension	22.75^a^	15.11	28.00^b^	14.66	30.64^b^	14.23	31.75^b^	11.74	43.15	< .001	.04 (.03-.05)

*Notes.* TGNC = transgender or gender non-conforming; SO = sexual orientation; *N* = 2,938; ADHD range = 0–54; Internalizing range = 0–63; For partial eta squared, .01 to .05 is a small, .06 to .13 is a medium, and > = .14 is a large effect

For these exploratory analyses, we again conducted two one-way univariate analyses of variance tests (i.e., 4 × 1 ANOVAs) to examine S/G/SO differences in ADHD and internalizing symptoms. For ADHD symptoms, the mean for the cisgender and heterosexual group was 16.88 (*SD* = 11.60), the mean for the cisgender and gay/lesbian group was 21.92 (SD = 11.84), the mean for the cisgender and bisexual group was 22.10 (*SD* = 11.67), and the mean for the TGNC group was 23.48 (*SD* = 11.12). This ANOVA was statistically significant (*F* = 36.28, *p* < .001, partial η^2^ = 0.04) and corresponded to a small effect. Again, we conducted post-hoc Games-Howell analyses; all three post-hoc paired comparisons between the cisgender/heterosexual group and each of the SGM groups were significant (*p*s < .001) such that SGM participants reported higher levels of ADHD symptoms than their cisgender/heterosexual peers. None of the comparisons among the three SGM groups were significant.

For internalizing symptoms, the mean for the cisgender and heterosexual group was 22.75 (*SD* = 15.11), the mean for the cisgender and gay/lesbian group was 28.00 (SD = 14.66), the mean for the cisgender and bisexual group was 30.64 (*SD* = 14.23), and the mean for the TGNC group was 31.75 (*SD* = 11.74). This ANOVA was also statistically significant (*F* = 43.15, *p* < .001, partial η^2^ = 0.04) and corresponded to a small effect. Again, all three post-hoc Games-Howell paired comparisons between the cisgender/heterosexual group and each of the SGM groups were significant (*p*s < .001) such that SGM participants reported higher levels of internalizing symptoms than their cisgender/heterosexual peers.^2^ None of the comparisons among the three SGM groups were significant.

## Discussion

In psychopathology research, we have historically ignored sex/gender and conducted sex- and gender-neutral research (Hartung & Lefler, 2019; Howard et al., 2017; Lefler et al., 2023). In most studies, we do not analyze sex, gender, or sexual orientation. For studies in which sex/gender are analyzed, a binary approach is almost always used (Hyde et al., 2019; Keyes & Platt, 2023). The goal of the current study was to compare several different approaches to conducting psychopathology analyses with S/G/SO in an emerging adult sample.

When two-group (i.e., binary) analyses were conducted, with both sex and gender, there was no significant between-group difference for ADHD symptoms. Although ADHD has traditionally been shown to be more common in boys and men, our results are consistent with more recent studies suggesting that the sex ratio in adults is closer to 1:1 (Hinshaw et al., 2022), and this is especially true when examining symptom levels, rather than prevalence rates, in emerging adult and college samples (e.g., Fedele et al., 2012). Next, regarding the two-group analyses for internalizing symptoms (with both binary sex and gender identity analyses), females/women reported higher levels than males/men. This finding is consistent with past research suggesting that women are more likely to be diagnosed with depression and anxiety, and that they report higher levels of depression symptoms (Keyes & Platt, 2023; McLean et al., 2011; Salk et al., 2017).

When three-group analyses were conducted, we found that the SGM group reported significantly higher levels of both ADHD and internalizing symptoms, and there was no difference between cisgender-heterosexual (cis-hetero) men and women. For both symptom dimensions, the SGM group was significantly higher than both the CHW and CHM with medium effects. Although research has demonstrated that SGM individuals report higher levels of anxiety and depression than their cis-hetero peers (Fish & Pasley, 2015; Kattari et al., 2020; King, et al., 2008), there has been very little research on ADHD in SGM individuals. For example, in a systematic review of neurodevelopmental disorders in individuals with gender dysphoria, Thrower et al. (2020) found that autism spectrum disorders occur more frequently in this group; however, they did not find sufficient studies examining ADHD.

In the current study, the differences between the SGM and cis-hetero groups appeared to be practically and clinically significant. For example, the mean severity of ADHD symptoms for the SGM group was 22.19, and the means for the CHW and CHM groups were 15.93 and 16.72, respectively. These severity scores were based on a 4-point Likert scale (0 = *Never or rarely* to 3 = *Very often*). Clinically, a score of 2 (*Often*) or 3 (*Very often*) is typically interpreted to mean that the symptom is present. Thus, a mean of 22.19 would be consistent with endorsing 11 of 18 symptoms as occurring *often* or 7 of 18 symptoms as occurring *very often*; whereas a mean of 15.93 would be consistent with endorsing 8 of 18 symptoms as occurring *often* or 5 of 18 symptoms as occurring *very often.* Thus, individuals in the SGM group are endorsing 2 to 3 more symptoms, on average, than members of either cis-hetero group. Given that a diagnosis of ADHD requires 5 of 9 symptoms of inattention and/or 5 of 9 symptoms of hyperactivity/impulsivity in adulthood, and diagnostic decisions often come down to just a symptom or two, this difference is clinically significant. Although our INT measure does not lend itself to a symptom analysis, the magnitude of the differences for ADHD and INT were similar. Specifically, those in the SGM group reported 33–39% more ADHD and 37–44% more INT than either cis-hetero group.

These findings for the SGM group are consistent with minority stress theory, which would suggest that individuals in the SGM group reported higher levels of ADHD and INT symptoms because of the additional stress that they experience in our society as members of this group. Although we expected this finding for INT, we were surprised at the magnitude of the difference in a highly heritable, neurodevelopmental disorder like ADHD (Brikell et al., 2015). Additional analyses showed that our findings held for ADHD when we controlled for INT and vice versa. Nonetheless, future research (and clinical work) should examine, via clinical interviews, whether our participants might be endorsing ADHD symptoms that would be better accounted for by other factors (e.g., endorsing difficulty paying attention as an ADHD symptom which may actually be due to anxiety, depression, or stress; endorsing disorganization as an ADHD symptom which may be caused by general cognitive overload).

Another finding that was surprising was that there were no sex/gender differences between CHW and CHM for INT symptoms. Higher rates of anxiety and depression diagnoses and symptoms in women is a well-established finding (McLean et al., 2011; Salk et al., 2017). The differences between our two- and three-group findings for INT suggest that three-group analyses are potentially important. As expected, females/women reported higher levels of INT symptoms than males/men when binary analyses were conducted. However, in the three-group analyses, the SGM group was statistically and meaningfully significantly higher than either of the cis-hetero groups, and the differences that were previously attributed to binary sex/gender disappeared. Given that a higher percentage of biological females identify as SGM than biological males (Lefler et al., 2023), it is possible that higher rates of INT in SGM have been misattributed to sex/gender. However, given that identification with SGM status is increasing generationally, with individuals in younger cohorts being much more likely to identify as SGM than individuals in older cohorts, it is unlikely that higher rates of INT in females/women would have completely been accounted for by SGM historically. This certainly warrants more research attention.

Given that our three-group analyses produced statistically and clinically significantly higher endorsement of ADHD and INT in the SGM group and that we had a large enough sample, we conducted additional exploratory analyses with four groups to determine whether there were any differences among more specific SGM identities. We found that all three SGM groups (i.e., cisgender + gay/lesbian, cisgender + bisexual, and TGNC) reported significantly higher levels of ADHD and INT than their cis-hetero peers with small to medium effects. Furthermore, there were no statistically significant differences among the three SGM groups. Thus, minority stress may be impacting gay/lesbian, bisexual, and TGNC individuals at similar levels, though more research is needed to confirm this, especially given the paucity of research on and the anti-transgender laws that target TGNC individuals.

### Limitations and Future Directions

As with any paper, the current study had limitations that should be considered. First, it is important that researchers consider not only S/G/SO more systematically but also variables related to race and ethnicity. It was beyond the scope of this project for us to also address race and ethnicity, but similar problems exist with this other set of demographic variables (Eaton, 2019). That is, race and ethnicity are often conflated as are sex and gender, and researchers tend to ignore these variables as they often do with sexual orientation (i.e., race/ethnicity are more often included in participants sections to describe the sample than are sex/gender/sexual orientation; however, similar to the latter, they are rarely analyzed; Eaton, 2019). In the current study, we focused on S/G/SO and examined several approaches to analyzing these individual difference variables. Future research aimed at doing the same for race and ethnicity variables is certainly warranted, especially given the disparities in people of color accessing mental health services (Lu et al., 2021).

Second, even in our attempts to analyze groups beyond the binary (i.e., our three- and four-group analyses), we still grouped participants together in ways that have limitations. For example, in our three-group analyses, we grouped all SGM individuals together in a way that might suggest that they are a homogeneous group when they certainly are not. Likewise, in our four-group analyses, we separated the SGM individuals into three groups, but we still grouped all cis-gender gay and lesbian participants and all TGNC participants together. This might ignore important nuances, such as whether transwomen have substantially different experiences from transmen or how trans individuals who “pass” might be impacted, both of which are certainly areas for further study. While we maintain that these groupings are superior to ignoring SGM participants and their experiences, we understand that some nuance is lost. In the future, researchers should over-select for specific SGM subpopulations.

Next, in the current study we focused on only two sets of symptoms (i.e., ADHD and internalizing), used only online self-report of these symptoms in a community sample, and did not assess diagnosis. While we made these choices to provide a simple demonstration of our point regarding S/G/SO analyses with a large sample, we understand that these are limitations. We are unable to comment on how this would play out with evidence-based assessment of ADHD, depression, and anxiety, and we recognize that self-report of symptoms in a quick, online survey may be suspect. For instance, a person in our sample may have endorsed symptoms such as frequent worry and poor concentration for a myriad of reasons. Future researchers may want to test our analytic approach in other ways.

Finally, we acknowledge that the terminology in this area is quickly changing. While we attempted to use up-to-date terms, we recognize that these may be out of date in just a matter of months. For example, when we designed the study we used “biological sex” as a category instead of “sex assigned at birth.” In the intervening months we have learned more and have updated our own language, and we will continue to do so. We encourage researchers to carefully consider the language in their demographics sections with an emphasis on inclusion, and we point to several excellent resources (Beischel et al., 2022; Broussard et al., 2018; Hyde et al., 2019; Lowik et al., 2022).

### Research Recommendations

We recommend that psychopathology researchers use a three-group approach (i.e., including an SGM group) when sample size allows. This may change conclusions about binary sex differences, including those that are well-established in the literature. The utility of analyzing data with a separate SGM group has become even more pertinent given that more individuals are identifying as SGM in younger generations (Lefler et al., 2023). We also recommend that researchers conduct preliminary analyses with S/G/SO. These preliminary analyses, and possible follow-up analyses, should be conducted even if there is not a specific hypothesis related to S/G/SO. The goal is to determine whether results generalize across S/G/SO and to avoid sex- and gender-neutral research. When the preliminary analyses show no differences by S/G/SO on study variables, then no additional analyses are needed; however, researchers should note whether there was enough power to rule out S/G/SO differences.

When the preliminary analyses show S/G/SO differences, then researchers should consider whether to: (a) include S/G/SO as an independent, mediator, or moderator variable in their primary analyses (covarying lacks ecological validity and erases group identities) or (b) conduct exploratory analyses with S/G/SO. Finally, if S/G/SO from primary or exploratory analyses are significant, researchers should discuss these results using context from previous findings in the literature which may not have been reviewed in the introduction due to the lack of specific hypotheses about S/G/SO. Finally, if S/G/SO analyses demonstrate SGM differences, researchers should consider analyses that divide this group into more specific groups (e.g., sexual minorities, gender minorities) if they have a large enough sample to do so.

Insufficient power to detect potential differences is an important consideration, and may preclude three-group analyses. Thus, when sample sizes are small, as is often the case in clinical studies, we recommend: (a) describing S/G/SO in the participants section, (b) conducting preliminary analyses with biological sex and gender identity for males/females and men/women, respectively, and (c) mentioning the importance of examining the findings in SGM group(s) as a limitation and future direction. If preliminary analyses show sex or gender differences, then we recommend the procedure described above.

### Overall Conclusion

In the current study, we demonstrated the importance of analyzing psychopathology data by sex, gender, and sexual orientation. In a large community sample of emerging adults who self-reported ADHD and internalizing symptoms, we found increasing levels of complexity and nuance at each level of data analysis. Specifically, our data had very little nuance when we ignored sex and gender, more when we analyzed using binary sex and gender groups, and even more when we considered SGM status. In conclusion, the three-group approach, although not ideal in terms of lumping sexual and gender minorities, is superior to the traditional sex- and gender-neutral and binary approaches. Furthermore, the three-group approach may represent current best practice given that: (a) few samples will have a large enough gender minority group to separate from the sexual minority group and (b) these preliminary results did not show differences between sexual and gender minorities.

## Footnotes

1 For this paper, we will use both the terms SGM (NIH Sexual & Gender Minority Research Office, 2024) and LGBTQ+to refer to people who identify as lesbian, gay, bisexual, asexual, transgender, Two-Spirit, queer, and/or intersex, etc. We recognize that language is constantly evolving, and we strive to use accurate, appropriate, and affirming terminology.

2 We repeated all analyses (i.e., Tables 1-4) while controlling for the other DV (i.e., controlled for INT in the ADHD analyses and controlled for ADHD in the INT analyses). For INT symptoms, the results always remained the same in terms of significance and effect size. For ADHD, there were two instances where the ANOVA became significant (i.e., both binary analyses) and two instances where the results stayed the same. Thus, the females/women being higher on ADHD is not a result of comorbid internalizing symptoms.
